# Central Nervous System Infection by Free-Living Nematode *Cephalobus cubaensis* in a Human Host in Africa

**DOI:** 10.3390/tropicalmed10020037

**Published:** 2025-01-28

**Authors:** Charlotte Sriruttan-Nel, Chelline Cairns, Shareen Boughan, Bhavani Moodley, Lisa Ming Sun, Wai Yin Chan, Arshad Ismail, Absalom Mwazha, Praniel Bennimahadeo, Nithendra Manickchund, Mthabisi Moyo, Thabani Nkwanyana, Mpumelelo Z. Msimang, Ahmed Essa, John Frean, Mahomed-Yunus Moosa

**Affiliations:** 1 Parasitology Reference Laboratory, Centre for Zoonotic Emerging and Parasitic Diseases, National Institute for Communicable Diseases, Johannesburg 2192, South Africa; shareenb@nicd.ac.za (S.B.); bhavanim@nicd.ac.za (B.M.); lisas@nicd.ac.za (L.M.S.); johnf@nicd.ac.za (J.F.); 2 School of Pathology, Faculty of Health Sciences, University of the Witwatersrand, Johannesburg 2193, South Africa; 3 Department of Infectious Diseases, Victoria Mxenge Hospital, Division of Internal Medicine, Nelson R Mandela School of Medicine, University of KwaZulu-Natal, Durban 4013, South Africa; cairnsc1@ukzn.ac.za (C.C.); muscleszn@yahoo.com (N.M.); moosay@ukzn.ac.za (M.-Y.M.); 4 Sequencing Core Facility, National Institute for Communicable Diseases a Division of the National Health Laboratory Service, Johannesburg 2192, South Africa; annie.chan@witsdih.ac.za (W.Y.C.); arshadi@nicd.ac.za (A.I.); 5 Department of Biochemistry, Genetics and Microbiology, Forestry and Agricultural Biotechnology Institute, University of Pretoria, Pretoria 0002, South Africa; 6 Department of Biochemistry and Microbiology, Faculty of Science, Engineering and Agriculture, University of Venda, Thohoyandou 0950, South Africa; 7 Department of Anatomical Pathology, National Health Laboratory Service, Durban 4001, South Africa; absalom.mwazha@nhls.ac.za (A.M.); mpumelelo.msimang@nhls.ac.za (M.Z.M.); 8 Discipline of Anatomical Pathology, Nelson R Mandela School of Medicine, School of Laboratory Medicine and Medical Sciences, University of KwaZulu-Natal, Durban 4041, South Africa; 9 Department of Diagnostic Radiology, Victoria Mxenge Hospital, Nelson R Mandela School of Medicine, University of KwaZulu-Natal, Durban 4013, South Africa; shaun46123@gmail.com; 10 Ear, Nose and Throat Surgery Department, Nelson R Mandela School of Medicine, University of KwaZulu-Natal, Durban 4041, South Africa; mthamoyodr.mm@gmail.com; 11 Department of Internal Medicine, Victoria Mxenge Hospital, Nelson R Mandela School of Medicine, University of KwaZulu-Natal, Durban 4013, South Africa; thabani_s@yahoo.com; 12 Histopathology Division, Lancet Laboratories, Durban Branch, Johannesburg 2090, South Africa; 11essa11@gmail.com; 13 Wits Research Institute for Malaria, Faculty of Health Sciences, University of the Witwatersrand, Johannesburg 2193, South Africa

**Keywords:** free-living nematode, *Cephalobus cubaensis*, central nervous system, meningoencephalitis

## Abstract

Background: Human central nervous system infections due to free-living nematodes, although extremely rare, are usually fatal. Immunodeficiency has not been a feature of most of these cases, unlike the situation pertaining to disseminated *Strongyloides stercoralis* infection. Case report: An elderly immunocompetent man presented with a history of tinnitus and otalgia, progressing to central nervous system involvement with confusion, weakness, and other neurological signs. Examination revealed a unilateral external auditory canal soft tissue mass and radiological evidence of ipsilateral temporal bone destruction and brain parenchymal disease. A biopsy of the ear canal mass revealed the presence of an unidentified nematode species, and treatment with anthelminthics was started. The patient’s clinical condition deteriorated and he died shortly after admission to the intensive care unit. The immediate cause of death was bronchopneumonia. During the autopsy, an extensive involvement of the right middle cranial fossa was found, with destruction of the squamous and petrous parts of the temporal bone. Results: We identified adult, larval, and egg stages of a free-living nematode in the antemortem external auditory canal tissue mass and the post-mortem brain samples. Polymerase chain reaction assays, with Sanger and whole-genome sequencing, identified *Cephalobus cubaensis*. This is a free-living species not previously known to be pathogenic to humans, although nematodes of the same genus have caused mastitis in horses. Conclusions: Microscopic appearance and the invasive behaviour of the pathogen evoked a putative diagnosis of *Halicephalobus gingivalis*, the most frequently reported free-living nematode infecting humans. However, this nematode’s size and anatomical features, and the clinical presentation and duration of illness, prompted the consideration of an alternative species. We speculate that an initial bacterial otitis externa provided the opportunity for colonization by the nematode from an environmental source and subsequent invasion.

## 1. Introduction

Nematodes are a phylum of non-segmented roundworms. They are among the most numerous and diverse metazoan organisms on the planet, comprising more than 27,000 species that inhabit soil, the aquatic environment, plants, and animals. Free-living nematodes are the majority and feed on environmental microorganisms; parasitic species can cause disease in various plants and animals. *Cephalobus cubaensis* is a free-living nematode belonging to the Order Rhabditida, Family Cephalobidae [1]. Cephalobs (members of this family) include a large number of nematodes, which are distributed worldwide. They are abundant inhabitants of soil where they feed on bacteria. Species within this family display variation in morphology; however, challenges in characterization have resulted in the use of molecular techniques for identification [2]. Most *Cephalobus* species have only female worms and are assumed to be parthenogenetic [2]. Infection in vertebrates is extremely rare, with only two reports described in animals; verminous mastitis in mares due to *Cephalobus* species has been reported in the United States of America and South Africa [3,4]. No previous human cases of cephalob infection have been reported.

### Case Report

**Figure 1:**
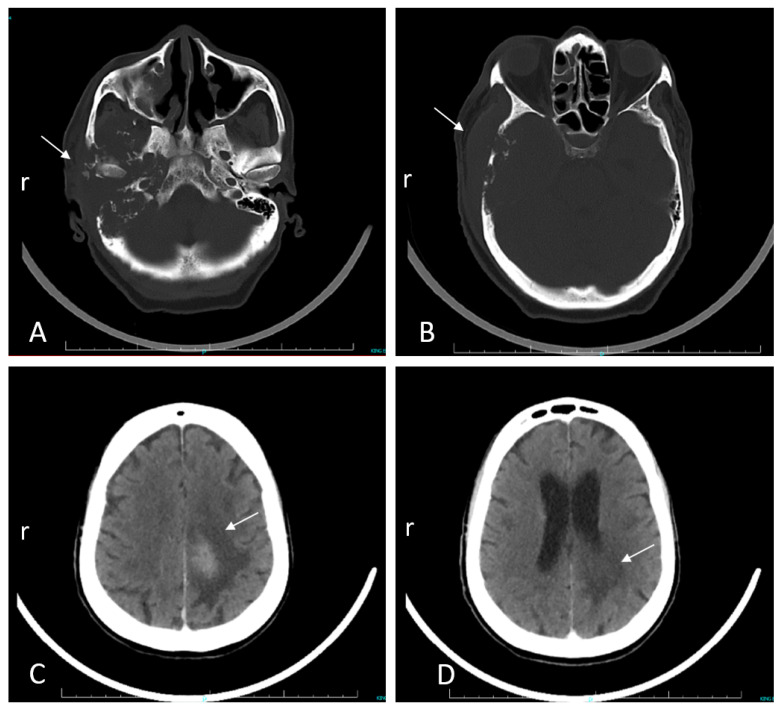
Antemortem computed tomography (CT) brain scans. Arrows indicate respective lesions. (**A**) Non-enhanced CT showing bony destruction and erosion of the right-side mastoid bone and facial nerve canal, the petrous and squamous temporal bones, and zygomatic process. (**B**) Non-enhanced CT at orbital cavity level showing extracranial soft tissue swelling and temporal bone erosion. (**C**) Contrast-enhanced CT demonstrating the hyperdense left parietal parenchymal brain lesion with surrounding edema that extends inferiorly, shown in (**D**).

**Figure 2:**
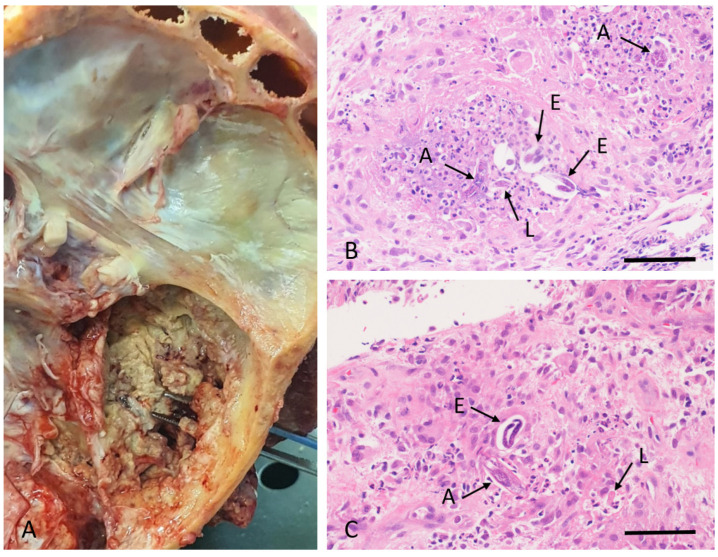
Pathological features at autopsy. (**A**) Extradural necrotic material in the right side of the middle cranial fossa base and external auditory meatus. (**B**,**C**) Histopathological images of brain tissue, showing nematode adults (A), larvae (L), and eggs (E) in various section planes (arrows) and a mixed inflammatory infiltrate (H&E stain; bars = 100 µm).

In August 2022, a clergyman in his mid-seventies travelled from the Democratic Republic of Congo to Durban, South Africa, to seek medical care, having developed otalgia and tinnitus eight months prior. He had been well previously, did not use tobacco or alcohol, and had no known history of skin, ear, or eye problems. There had been no exposure to farm animals or pets. Initial symptoms were followed by a growing mass in the external auditory canal, swelling in the temporomandibular region, otorrhoea, hearing loss, and right facial muscle weakness. Over the three months prior to admission, he developed confusion, drowsiness, slurred speech, eating difficulties, urge incontinence, and inability to walk. An examination showed mild generalized wasting, but no obvious pallor, no head and neck lymphadenopathy, and no skin lesions. A right-side lower motor neuron seventh nerve palsy was present. All other cranial nerves were intact. Otorhinolaryngological examination found a trismus, a prominent firm swelling over the right temporomandibular area, and an exophytic fleshy mass in the ipsilateral external auditory canal, with a serosanguinous discharge (Video S1, Supplementary Information). Laboratory investigations revealed microcytic anemia (hemoglobin of 8.8 g/dL) [normal range: 12–15 g/dL] and mild eosinophilia of 0.53 × 10^9^/L [normal range: 0–0.5 × 10^9^/L]. Computed tomography of the brain showed erosions of the right temporal bone with a soft tissue mass extending through the middle ear into the external auditory canal (Figure 1A,B). There was also a minimally enhancing 3.1 cm diameter hyperdense lesion with surrounding vasogenic edema in the left parietal lobe (Figure 1C,D). The differential diagnoses included temporal bone squamous cell carcinoma and lymphoma. Other malignancies or cholesteatoma were thought to be less likely. The infectious causes considered were necrotizing otitis externa, tuberculosis, and fungal skull base osteomyelitis. Histopathology of tissue biopsies of the ear mass revealed an inflammatory background with granuloma formation, central microabscesses, and associated nematode larvae, possibly *Strongyloides stercoralis*. Bone destruction by the inflammatory process was observed, without evidence of malignancy. Lumbar puncture revealed a normal opening pressure and clear colourless cerebrospinal fluid (CSF), with an elevated protein (0.83 g/L [normal range: 0.15–0.45 g/L]), normal glucose (3.9 mmol/L [normal range: 2.5–4.4 mmol/L]), lymphocyte count of 44/µL (normal range: 0–5/µL), erythrocyte count of 90/µL (normal range: <1/µL), and no polymorphonuclear cells. No organisms were observed on Gram-stain and bacterial culture was negative. C-reactive protein was 69 mg/L (normal range: <10 mg/L), and glycated hemoglobin was 5.4% (normal range: <5.7%). HIV-1/2 ELISA and HTLV-1 serology were negative. Cerebrospinal fluid, auditory canal fluid, and fresh tissue from the biopsied ear mass were referred to the Parasitology Reference Laboratory (PRL) at the National Institute for Communicable Diseases (NICD) for further investigation. Empiric treatment included albendazole (400 mg bd) and intravenous methylprednisone (750 mg daily for three days), followed by dexamethasone (8 mg daily IVI). The level of consciousness declined. On day six of admission, praziquantel (1500 mg tds) and oral ivermectin (18 mg daily) were added. Subsequently, the patient developed respiratory failure and died after transferring to the Intensive Care Unit for ventilatory support. At autopsy, an external examination showed the right temporomandibular area swelling with necrotic material in the external auditory canal. Further dissection revealed extradural necrotic tissue involving the middle and inner ear with erosion of the petrous and squamous parts of the temporal bone extending externally, eroding the mastoid portion of the temporal bone (Figure 2A). Microscopic examination of the brain and meninges showed extensive necrosis, inflammation, and nematodes primarily affecting the left temporal lobe and to a lesser extent, the right temporal lobe, pons, and cerebellum. Meningoencephalitis, with perivascular inflammation comprising lymphocytes and macrophages, was present. Adult female nematodes, larvae, and eggs surrounded by a granulomatous inflammatory response were observed within the brain parenchyma, some within blood vessels (Figure 2B,C). The necrotic material from the right temporomandibular region and brain biopsies showed abundant nematodes. The spinal cord was not examined. The lungs showed bronchopneumonic changes. Nematodes were not seen in any other organs. The cause of death and final diagnosis was that of progressive parasitic meningoencephalitis with terminal bronchopneumonia.

## 2. Materials and Methods

### 2.1. Laboratory Methods

#### Samples

Samples received at PRL for microscopy, culture, and molecular analysis included antemortem CSF and formalin-fixed paraffin-embedded and unfixed tissue biopsies from ear mass and ear canal fluid. Autopsy specimens included inner ear mass and brain tissue.

### 2.2. Microscopy

We performed light microscopy on preparations of ear discharge fluid, CSF, and ear tissue mass. Direct wet preparations were viewed under low-power magnification (100×). Giemsa-stained preparations were examined at low and higher (500×, 1000×) magnifications. Routine histopathopathology tissue sections stained with H&E were viewed.

### 2.3. Nematode Culture

Samples were planted on blood and malt extract agar plates with lawns of *E. coli* (ATCC 25922) and incubated at 30 °C. Antibiotics were added to inhibit *Proteus* sp. bacterial contamination. Minimum Essential Medium (MEM) (ThermoFisher, Johannesburg, South Africa) with and without gentamicin (0.5 µg/mL), streptomycin (0.1 µg/mL), and ampicillin (50 µg/mL) (Merck, Johannesburg, South Africa) were also inoculated. Cultures were monitored microscopically.

### 2.4. PCR Assays

DNA was extracted from all samples except ear tissue, using the QIAamp DNA mini kit (Qiagen, Hilden, Germany). *Strongyloides stercoralis* PCR targeting a non-coding repeat region was initially performed [5], followed by nested PCR using primers targeting the large nuclear subunit ribosomal RNA gene of cephalob nematodes [6] (Table S1, Supplementary Information).

### 2.5. Sanger Sequencing

Amplicons from the cephalob PCR were purified using the QIAquick Gel Extraction Kit (Qiagen, Hilden, Germany) and submitted for Sanger sequencing (Inqaba Biotec, Pretoria, South Africa). Raw data from all samples were edited and cleaned to generate a consensus sequence (BioEdit, Informer Technologies, Los Angeles, CA, USA).

### 2.6. Whole-Genome Sequencing

The ear mass biopsy sample was prepared using the NEBNext Microbiome DNA Enrichment Kit (New England Biolabs, Ipswich, MA, USA) to remove human DNA. Original and cultured samples were subjected to whole-genome sequencing. Paired-end libraries (2 × 150 bp) were prepared using the Illumina DNA Prep kit (Illumina, San Diego, CA, USA), followed by sequencing on the Illumina NextSeq 2000 platform (Illumina, San Diego, CA, USA). Raw sequence reads were processed with quality checks and removal of low-quality and adapter regions by Trim galore v0.6.2 (Babraham Bioinformatics, Cambridge, UK), followed by removal of human host reads using Bowtie2 v2.4.2 [7]. Remaining reads were used for de novo metagenome assembly with MetaSPAdes v3.14.1 [8]. The genome completeness of the targeted nematode was evaluated using benchmarking universal single-copy orthologs (BUSCO) [9].

### 2.7. Phylogenetic Analysis

The 18S and 28S rRNA sequences of species from the same nematode clade were accessed from the National Center for Biotechnology Information (NCBI). The rRNA sequences of the sample were extracted from the de novo metagenome assembly results using BAsic Rapid Ribosomal RNA Predictor version 0.9 https://github.com/tseemann/barrnap (accessed on 17 October 2022). The 18S rRNA sequence alignment (1759 bp) was used for maximum likelihood phylogenetic tree construction with RAxMLv8.2.12, [10] with 1000 bootstrap and GTRGAMMAX model. The partial sequence of the 28S rRNA sequence alignment (1080 bp) was used for maximum likelihood phylogenetic tree construction using IQ-TREE v.2 [11] with 1000 bootstrap and GTR+I+G model.

## 3. Results

### 3.1. Microscopy

**Figure 3:**
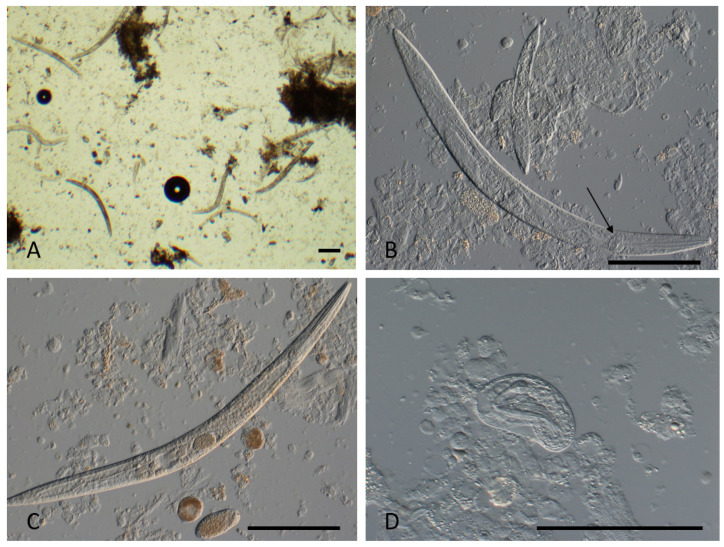
Free-living *Cephalobus cubaensis* nematodes in ear canal exudate sample. (**A**) Low-power view, showing adults, larvae, and eggs, standard illumination. (**B**) Adult female and larval stages, interference contrast illumination. Note the rhabitiform single-bulb esophagus (arrow). (**C**) Adult female stage, with egg in uterus; free eggs in the exudate. (**D**) Embryonated egg. All bars = 100 µm.

All tissue samples showed the presence of nematodes. Serous fluid (negative for beta-2-transferrin, therefore excluding CSF) from the auditory canal demonstrated nematode adults, larvae, and ova on microscopy (Figure 3). The rhabditiform morphology of the larvae initially suggested *Strongyloides* or *Halicephalobus* species. Adult nematodes were measured to be 475 to 487 μm by 34–40 μm, showing a smooth cuticle, a rhabditiform esophagus, and a female genital tract; however, the absence of a dorsiflexed ovary and double-bulbed esophagus, and the measured size of all stages, indicated a different free-living nematode species than first thought. The larvae were measured to be 166 to 417 μm to 10–32 μm, and the thin-walled, round to oval eggs were measured to be 50–57 μm by 21–29 μm.

### 3.2. Culture

Motile larvae and ova were observed microscopically on the blood agar plate after three days’ incubation (Video S2, Supplementary Information). There was an overgrowth of *Proteus* sp., which potentially led to the failure of subsequent culture attempts.

### 3.3. Molecular Identification and Confirmation

#### 3.3.1. PCR and Sanger Sequencing

For all samples, the *S. stercoralis* PCR was negative and the cephalob PCR was positive. Sanger sequencing results on ear tissue and ear canal fluid samples passed quality checks for editing. Since the entire PCR amplicon could not be sequenced due to its length (~1200 bp), forward and reverse sequences were concatenated to generate one fragment. BLAST analysis showed alignment with regions 21–275 and 766–981 of the 28S ribosomal RNA gene of *Cephalobus cubaensis* (GenbankDQ903102.1) with 99% query cover, 4 × 10^−120^ E-value, and percentage identity of 98.06%.

#### 3.3.2. Whole-Genome Sequencing

One hundred and eighty million paired-end raw reads were generated. After data trimming and removal of host sequences, a 13× sequencing coverage for the 18S rRNA and 28S rRNA gene sequences was obtained. However, only 6.2% completeness of the genome was retrieved based on BUSCO v5.2.2 assessment, using the eukaryote odb10 dataset. From the combined data analysis, the 18S rRNA (1660 bp) sequence similarity was 98.83% to *C. cubaensis* with 96% BLAST query coverage, and the 28S rRNA (4029 bp) sequence had a similarity of 92.09% to *Zeldia punctata* with 85% query coverage (891 bp–4002 bp). Part of the 28S rRNA sequence (1612 bp–3510 bp) showed a 93.72% similarity to the partial 28S rRNA gene region of *Heterocephalobellus* sp. JB-8, and another partial region of the 28S rRNA (489 bp–1487 bp) showed a higher 95.80% similarity to the partial 28S rRNA gene of *C. cubaensis* strain PS-1197 (Table 1).

#### 3.3.3. Phylogenetic Analysis

**Figure 4:**
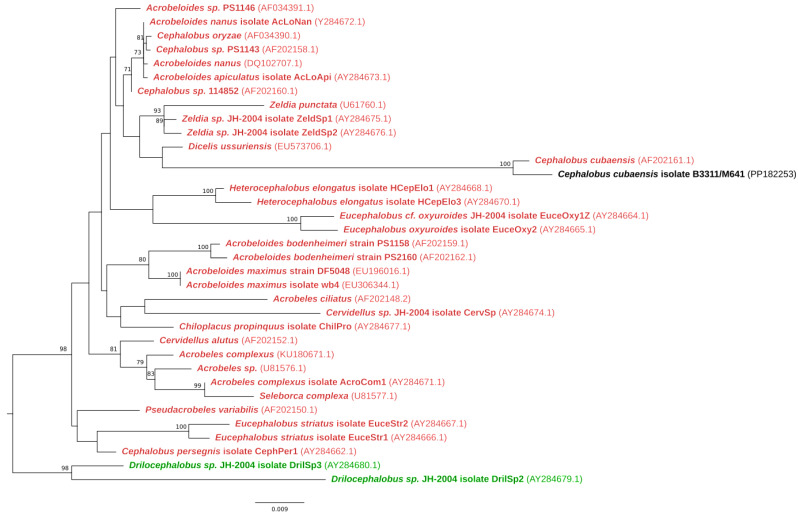
Maximum likelihood 18S rRNA phylogenetic tree showing *Cephalobus cubaensis’* relationship to related species with bootstrap support above 70% (GenBank accession numbers in parentheses). The isolate sample in this study is in black text. The scale bar represents the inferred substitutions per nucleotide position. Outgroup is *Drilocephalobus* species (green text).

Based on the ML 18S and 28S rRNA phylogenetic tree results, the ear mass biopsy isolate (B3311/M641) grouped closely with the species *Cephalobus cubaensis* (Figure 4 and Supplementary Information).

## 4. Discussion

This report describes the first known human case of infection caused by a *Cephalobus* species globally and the first reported human case caused by any free-living nematode in Africa. This case is unique in that an antemortem diagnosis was rendered and antihelminthic treatment commenced. Invasive free-living nematode infections in humans are very rare. There have been only six such cases described over the past four decades, all attributable to *Halicephalobus* species [12,13]. All previous cases had fatal central nervous system involvement, with no pathogen-specific treatment commenced before death. *Halicephalobus* infections have been described in equines [6,14], with uniformly fatal central nervous system infections despite anthelminthic therapy. In humans, the diagnosis was confirmed by molecular testing in only two described instances. A single case of bilateral otitis externa associated with a free-living nematode, *Rhabditis* sp., was documented in Germany [15]. The origin of the helminth infection remained unclear, but as was possible in our case, bacterial otitis externa may have facilitated nematode superinfection. *Rhabditis* spp. nematodes have been reported in stool, urine, and vaginal swab samples, but their role as pathogens, as opposed to environmental contaminants, is typically obscure [15]. In our patient, the history did not provide clues to the mode of acquisition of infection, although localized exposure to environmental organic matter was likely. The unusual clinical presentation of an ear mass facilitated the tissue biopsy. The recognition of larvae in histopathological sections and access to a reference laboratory were key to establishing the diagnosis. Morphological features of larvae and molecular testing expedited the exclusion of *S. stercoralis* and *H. gingivalis*. The identification of the pathogen via whole-genome sequencing on tissue samples (ante- and post-mortem) was challenging. The 18S rRNA gene sequence results were the same across all sample types and showed high similarity and coverage of *C. cubaensis*. The 28S rRNA gene sequence had lower query coverage and specificity, indicating that this genomic region may be less useful for identification; however, these data also indicated *C. cubaensis* and other members of the Cephalobidae. It must be noted that only very few sequences of this species have been deposited in databases. The similarity between the few previously deposited 18S sequences was higher compared to the one described here, highlighting the limitation related to the small number of whole-genome sequencing reads. The failure to culture the nematode limited the availability of genomic material for analysis. To date, the most comprehensive phylogenetic analysis of nematodes within the family Cephalobidae [1] showed the utility of 18S and 28S rRNA gene sequencing for species identification.

## Figures and Tables

**Table 1:** BLAST analysis results obtained from whole-genome sequencing.

Combined Data Analysis			
Gene	Species	Identity	BLAST Query Coverage
18S	*Cephalobus cubaensis*	98.83%	96%
28S	*Cephalobus cubaensis*	95.80%	25%
	*Heterocephalobellus* sp.	93.72%	47%
	*Zeldia punctata*	92.09%	85%

## Data Availability

The original contributions presented in this study are included in the article/Supplementary Materials. Further inquiries can be directed to the corresponding author.

## Supplementary Materials

The following supporting information can be downloaded at https://www.mdpi.com/article/10.3390/tropicalmed10020037/s1, Figure S1. Time-lapse images of adult worm laying egg. Figure S2. Maximum likelihood 28S rRNA phylogenetic tree showing *Cephalobus cubaensis’* relationship to related species with bootstrap support above 70% (GenBank accession numbers in parentheses). The isolate sample in this study is in black text. The scale bar represents the inferred substitutions per nucleotide position. Outgroup comprises *Cervidellus alutus* and *Acrobeles complexus* (green text). Table S1. Primer sequences for nested PCR targeting the large nuclear subunit ribosomal RNA gene of cephalob nematodes. Video S1. Otoscopy of the right external auditory canal, showing the exudative inflammatory tissue mass obstructing the canal. Video S2. External auditory canal exudate cultured on blood agar with *E. coli* lawn, showing an adult gravid female nematode moving on the surface.
