# Supplementary material for: Central Nervous System Infection by Free-Living Nematode Cephalobus cubaensis in a Human Host in Africa
Source: Trop Med Infect Dis. 2025 Jan 28;10(2):37. doi: 10.3390/tropicalmed10020037 (PMC11860163; doi:10.3390/tropicalmed10020037)
Supplement: Supplementary file 1 [file tropicalmed-10-00037-s001.zip › Table suppl. PCR primers.pdf]

Table S1. Primers used for nested PCR targeting the large nuclear subunit ribosomal RNA gene of cephalob nematodes

Primary PCR

#391 forward: 5’ - AGCGGAGGAAAAGAAACTAA - 3’

#501 reverse: 5’ - TCGGAAGGAACCAGCTACTA - 3’

Secondary PCR

#504 forward: 5’ - CAAGTACCGTGAGGGAAAGTTG - 3’

#503 reverse: 5’ - CCTTGGTCCGTGTTTCAAGACG - 3’
