# Supplementary figures and images for: Central Nervous System Infection by Free-Living Nematode Cephalobus cubaensis in a Human Host in Africa
Source: Trop Med Infect Dis. 2025 Jan 28;10(2):37. doi: 10.3390/tropicalmed10020037 (PMC11860163; doi:10.3390/tropicalmed10020037)

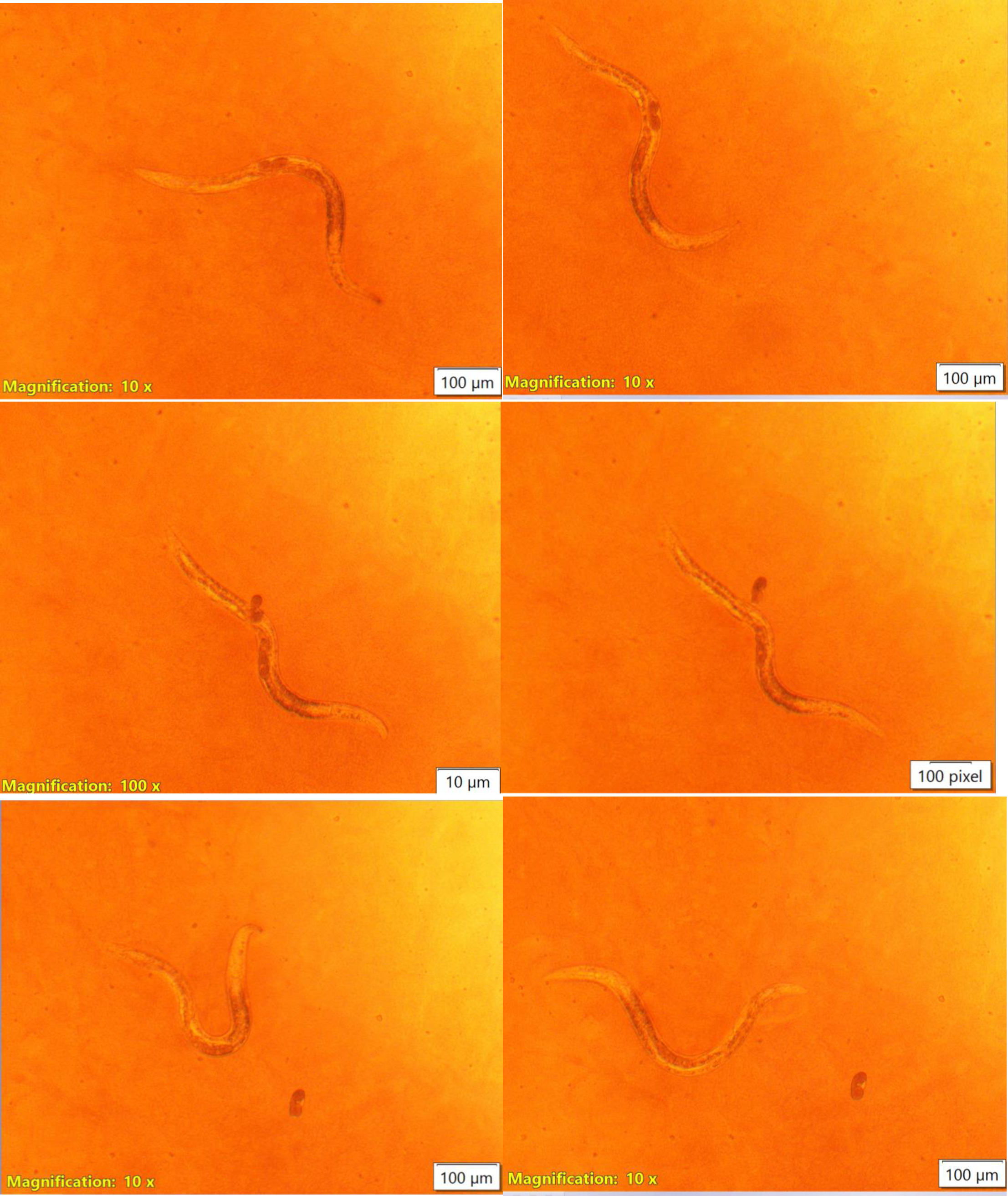

Supplement: Supplementary file 1 [file tropicalmed-10-00037-s001.zip › Figure s1.tif]

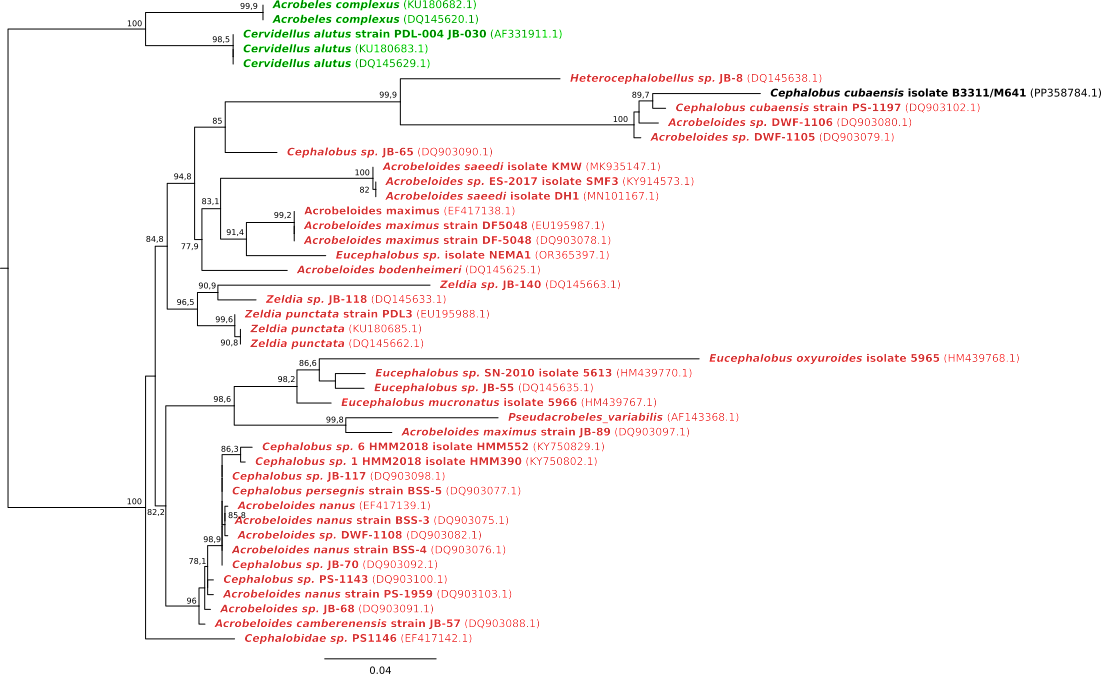

Supplement: Supplementary file 1 [file tropicalmed-10-00037-s001.zip › Figure s2.tif]
